# Supplementary material for: A high-resolution mRNA expression time course of embryonic development in zebrafish
Source: eLife. 2017 Nov 16;6:e30860. doi: 10.7554/eLife.30860 (PMC5690287; doi:10.7554/eLife.30860)
Supplement: Supplementary file 6. [file elife-30860-supp6.zip › biolayout-clusters-files/Cluster080.html]

Cluster080


# Cluster080: Detail

### Go to ZFA detail

## GO

| GO ID | Description | Domain | Annotated | Expected | Observed | Adjusted p-value | Genes | Ensembl IDs |
| --- | --- | --- | --- | --- | --- | --- | --- | --- |
| GO:0021538 | epithalamus development | biological\_process | 12 | 0.02 | 2 | 0.030 | tbx2b pax6a | ENSDARG00000006120 ENSDARG00000103379 |
| GO:0060219 | camera-type eye photoreceptor cell diffe... | biological\_process | 15 | 0.02 | 2 | 0.048 | tbx2b pax6a | ENSDARG00000006120 ENSDARG00000103379 |
